# First intermediate hosts of *Paragonimus* spp*.* in Vietnam and identification of intramolluscan stages of different *Paragonimus* species

**DOI:** 10.1186/s13071-018-2897-2

**Published:** 2018-05-30

**Authors:** Pham Ngoc Doanh, Luu Anh Tu, Hoang Van Hien, Nguyen Van Duc, Yoichiro Horii, David Blair, Yukifumi Nawa

**Affiliations:** 1 Institute of Ecology and Biological Resources, Viet Nam Academy of Science and Technology, Hanoi, Viet Nam; 2 Graduate University of Science and Technology, Viet Nam Academy of Science and Technology, Hanoi, Viet Nam; 3 Laboratory of Veterinary Parasitic Diseases, Faculty of Agriculture, University of Miyazaki, Center for Animal Disease Control, University of Miyazaki, Miyazaki, Japan; 4 College of Marine and Environmental Sciences, James Cook University, Douglas, Australia; 5 Tropical Diseases Research Centre, Faculty of Medicine, Khon Kaen University, Khon Kaen, Thailand

**Keywords:** First intermediate host, *Paragonimus*, *Sulcospira quangtriensis*, Triculinae

## Abstract

**Background:**

Members of the genus *Paragonimus* require at least three hosts in their life-cycles. The obligatory first intermediate hosts are freshwater snails. In Vietnam, although seven *Paragonimus* species have been recorded, the natural first intermediate hosts of almost all species have not been confirmed. The aim of this study was, therefore, to investigate snail hosts of *Paragonimus* species in Vietnam, and to identify *Paragonimus* species at intramolluscan stages.

**Methods:**

Freshwater snails were collected from streams in Yen Bai and Quang Tri Provinces, where high prevalences of *Paragonimus* metacercariae in crab hosts have been reported. Snails were morphologically identified and then examined individually for *Paragonimus* cercariae using shedding and crushing methods. Chaetomicrocercous cercariae, the morphological class to which *Paragonimus* cercariae belong, were collected for morphological description and molecular species identification by analyses of *ITS*2 sequences. The infected snail species were identified based on analyses of nucleotide sequences of the *cox*1 gene.

**Results:**

Three snail species were found to be infected with *Paragonimus* cercariae at low infection rates, ranging between 0.07–1.0%. The molecular analyses identified them as *Sulcospira quangtriensis* and 2 species of subfamily Triculinae. In a phylogenetic tree, these two triculine snails were related to the genera *Gammatricula* and *Tricula* with low posterior probabilities. Thus we named them as Triculinae sp. 1 and Triculinae sp. 2. Cercariae from the three snail species, *Sulcospira quangtriensis*, Triculinae sp. 1 and Triculinae sp. 2, were molecularly identified as *Paragonimus westermani*, *P. heterotremus* and *P. proliferus*, respectively. The cercariae of the three species are morphologically similar to each other, but their daughter rediae can be distinguished by the length of the intestine and the number of cercariae per redia. The rediae of *P. westermani* have a long intestine and each contain 6–8 cercariae. In contrast, those of *P. heterotremus* and *P. proliferus* have a short intestine and each redia contain 10–12 and 5–6 cercariae, respectively.

**Conclusions:**

Three snail species, *Sulcospira quangtriensis*, Triculinae sp. 1 and Triculinae sp. 2, serve as the first intermediate hosts of *P. westermani*, *P. heterotremus* and *P. proliferus*, respectively, in Vietnam. The length of the intestine of rediae and the number of cercariae per redia are valuable characteristics for distinguishing between larvae of these *Paragonimus* species.

## Background

Lung flukes of the genus *Paragonimus* (Family Paragonimidae) are parasites of mammals, commonly cats and canids, and occasionally humans. Members of this genus require at least three different hosts to complete their life-cycles [1]. The first intermediate hosts are freshwater snails in which the flukes develop through several stages (sporocyst, mother and daughter rediae, and cercariae). The cercariae belong to the microcercous type (subtype chaetomicrocercous), possessing an anterior stylet, a knob-like tail, spines on the body and tail, but lacking unicellular glands [2]. Mature cercariae escape from the snail hosts and penetrate suitable crab/crayfish hosts to develop into an infective stage, metacercariae. Definitive hosts become infected by eating these crabs/crayfish containing live metacercariae [1].

Regarding host specificity, *Paragonimus* flukes typically infect a wider spectrum of definitive and second intermediate hosts, whereas specificity for the first intermediate host is usually very strict and vary geographically [1, 3, 4]. Thus, identification of snail hosts in each endemic region is essential for understanding of routes of transmission.

In Vietnam, *Paragonimus* metacercariae of seven species have been reported in northern and central provinces: *P. heterotremus*, *P. westermani*, *P. bangkokensis*, *P. harinasutai*, *P. proliferus*, *P. vietnamensis* and *P. skrjabini* [5]. Of these, *P. heterotremus* and *P. westermani* are important causative agents for human paragonimiasis in Asian countries. The former species is commonest in northern provinces, and the latter in central provinces [5]. So far, there has been only one report of microcercous cercariae from “*Oncomelania*” snails in Vietnam, in 2002 [6], at a time when only metacercariae of *P. heterotremus* was found [6, 7]. The cercaria was, therefore, identified as *P. heterotremus* without molecular evidence [6]. However, subsequent surveys have revealed the presence of six additional *Paragonimus* species, and metacercariae of two to four species has been found in the same area [5, 8]. Moreover, the taxonomy of the “*Oncomelania*” snails in Vietnam has been revised and remains uncertain [9–11]. They were re-identified as a new genus *Pseudotricula* [9]. Since the name *Pseudotricula* had been assigned previously [10], Dang and Ho [11] renamed their genus as *Vietricula*. Liu et al. [12] considered that *Vietricula* snails are closely related to the genus *Gammatricula*. Thus, data on first intermediate hosts of *Paragonimus* in Vietnam are scant and unclear. The aim of this study was, therefore, to investigate snail hosts of *Paragonimus* spp. in Yen Bai and Quang Tri Provinces, where *Paragonimus* metacercariae are highly prevalent with the dominance of *P. heterotremus* and *P. westermani*, respectively [8]. In this survey, we found cercariae of three *Paragonimus* species, each in a different snail species. The host specificity and the morphological features of different *Paragonimus* species in the snail hosts are discussed herein.

## Methods

### Collection and examination of snails

**Fig. 1:**
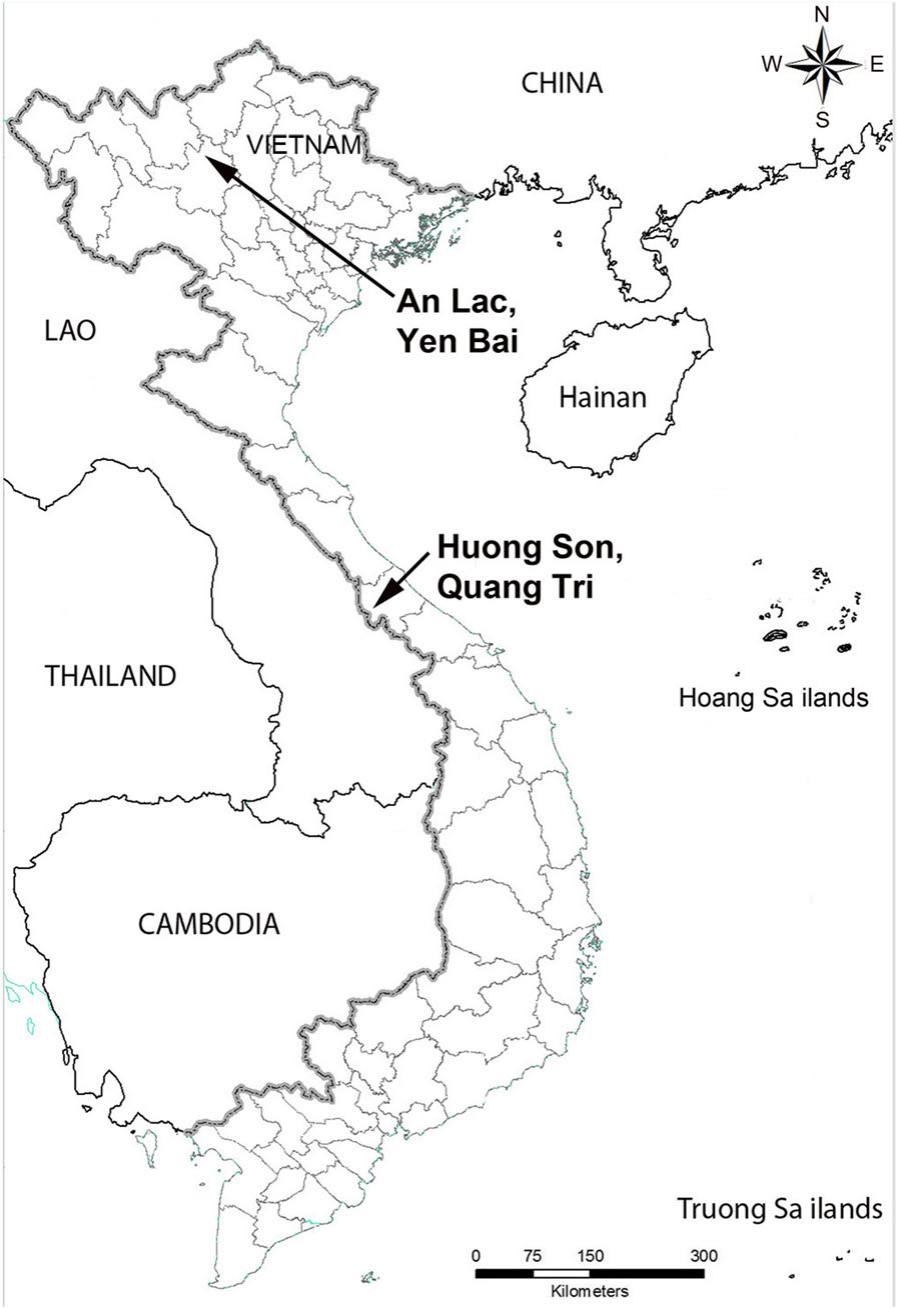
Geographical locations of sampling sites

Freshwater snails were collected in streams in An Lac Commune (Luc Yen District, Yen Bai Province) and Huong Son Commune (Huong Hoa District, Quang Tri Province) (Fig. 1) where high infection rates of crab hosts with *Paragonimus* metacercariae were found [8]. Snails were morphologically identified following Dang & Ho [13], and were examined for *Paragonimus* cercariae using shedding and crushing methods [14]. Snail samples were placed individually in 20 ml wide mouth plastic containers filled with 15 ml of water and left for 24 h. The containers were checked under a stereomicroscope at night and the next morning. If any chaetomicrocercous cercariae were found, they were used for morphological study. Afterwards, all snails were dissected under a stereomicroscope to identify cercariae that did not emerge, and other intramolluscan stages, such as rediae.

Live cercariae and rediae were observed under an Axio Lab A1 microscope (Carl Zeiss, Oberkochen, Germany). Photographs of the cercariae and rediae were taken using a Axiocam Erc 5s digital camera attached to a Axio Lab A1 microscope (Carl Zeiss, Oberkochen, Germany) and ZEN lite software (Carl Zeiss). Cercariae and rediae were drawn using Illustrator CS6. Measurements (in μm) of cercariae and rediae were taken. Live cercariae were identified according to Schell [2]. After morphological identification, a single cercaria and tissues from each infected snails were separately preserved in absolute ethanol for molecular identification of cercariae and snail hosts.

### Molecular analysis for identification of cercariae and snail hosts

Genomic DNA of chaetomicrocercous cercariae and snails from 10 infected snails was extracted using a PureLink Genomic DNA Mini Kit (Invitrogen, California, USA) following the manufacturer’s protocol. The internal transcribed spacer 2 (*ITS*2) region of nuclear ribosomal DNA from cercariae was amplified *via* polymerase chain reaction (PCR) with the primers 3S 5'-CGG TGG ATC ACT CGG CTC GT-3' as a forward primer and A28 5'-CCT GGT TAG TTT CTT TTC CTC CGC-3' as a reverse primer [15]. A portion of the mitochondrial cytochrome *c* oxidase 1 (*cox*1) gene of the infected snail species was amplified *via* PCR with the primers LCO1490 5'-GGT CAA CAA ATC ATA AAG ATA TTG G-3' as a forward primer and HCO2198 5'-TAA ACT TCA GGG TGA CCA AAA AAT YA-3' as a reverse primer [16]. PCR products were purified using a QIAquick PCR Purification Kit (Qiagen, California, USA) and sequencing reactions done using a Big-Dye terminator cycle sequencing kit v3.1 (Applied Biosystems, California, USA). Both forward and reverse strands were sequenced directly by a Genetic Analyzer (Model 3100, Applied Biosystems, California, USA), using the PCR primers as sequencing primers.

Sequences obtained from cercariae and snails were submitted to GenBank with accession numbers LC3605000-LC360505. Basic Local Alignment Search Tool (BLAST) searches in the server of the National Center for Biotechnology Information (NCBI) were used to determine the most similar sequences from named species. The *cox*1 sequences of the two triculine snail species showed low similarities to available sequences on GenBank. These sequences were therefore aligned with related sequences, and were used to reconstruct a phylogenetic tree using MrBayes [17]. The substitution model was selected using MrModelTest2 [18]. The model selected was the general time reversal (GTR) model with gamma-distributed rate variation across sites. The data were partitioned by codon position (first, second and third positions). Two parameters of the model (shape of the gamma distribution and substitution rates of the GTR model) were unlinked across partitions, to allow them to take different values. MrBayes was run for 2,000,000 generations, after which the standard deviation of split frequencies was below 0.01. Trees were sampled every 500 generations. ‘Burn-in’ was 25% of trees. A consensus tree was constructed, including all compatible groups of taxa.

## Results

### Identification of snails infected with chaetomicrocercous cercariae

The various snails collected belonged morphologically to *Melanoides*, *Sulcospira* and minute snails of the subfamily Triculinae. Snails of the genus *Melanoides* were few in number and clearly identified as *Melanoides tuberculata*. In contrast, *Sulcospira* snails were abundant, occurring at high densities, especially in larger rocky streams with high levels of water and fast flow. They usually attached to the surfaces of stones. The two species of triculine were commonly found attached to fallen leaves in small tributary streams with slow water flow.

Chaetomicrocercous cercariae were not found from *M. tuberculata* in either studied site or from *Sulcospira* sp. in Yen Bai Province, but were found from five *Sulcospira* sp. in Quang Tri, and from four and one triculine snails in Yen Bai and Quang Tri Provinces, respectively.

**Fig. 2:**
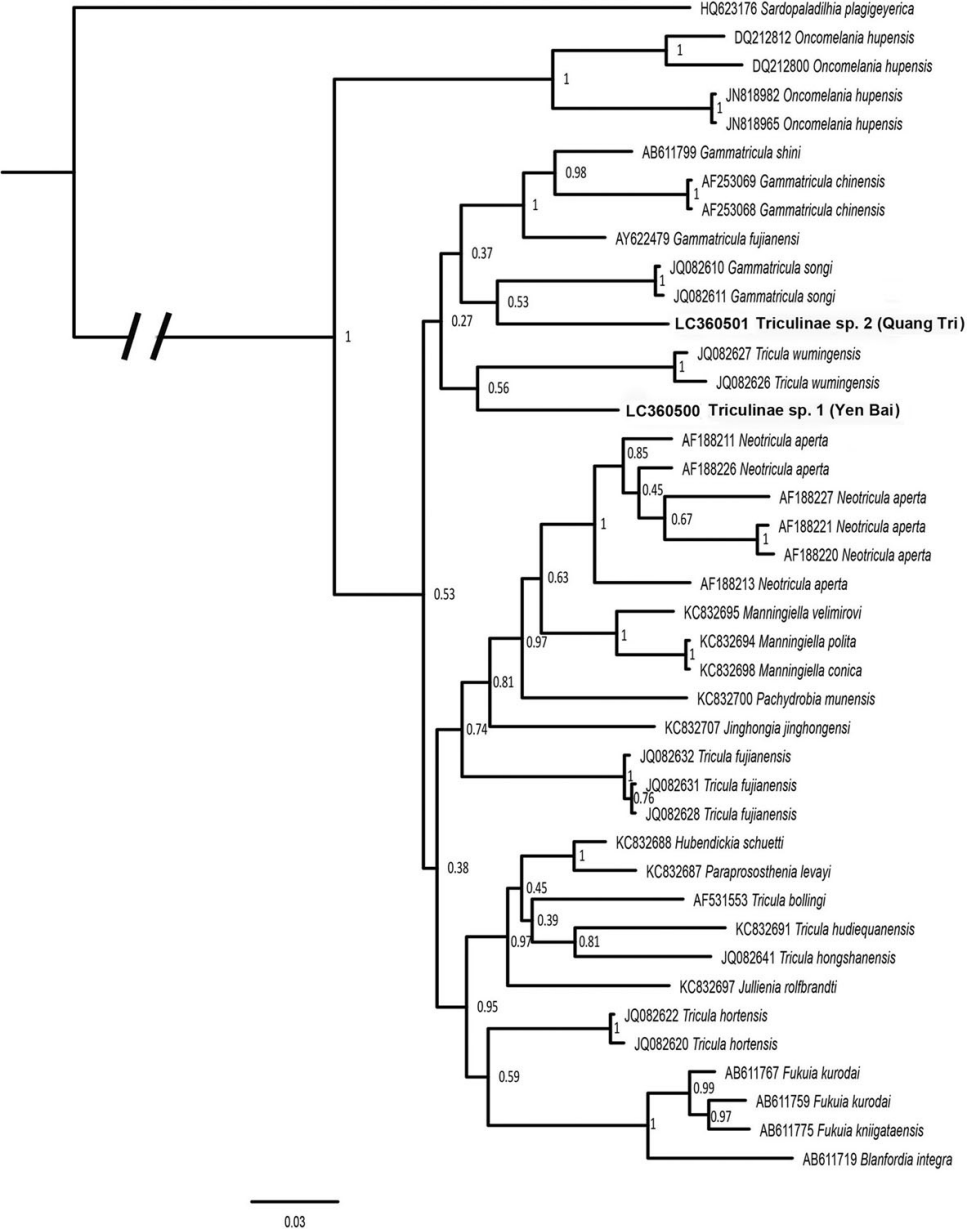
Phylogenetic tree reconstructed from *cox*1 sequences of triculine snails using Bayesian inference analysis. Numbers at nodes represent posterior probabilities. Samples obtained in this study are printed in bold

**Fig. 3:**
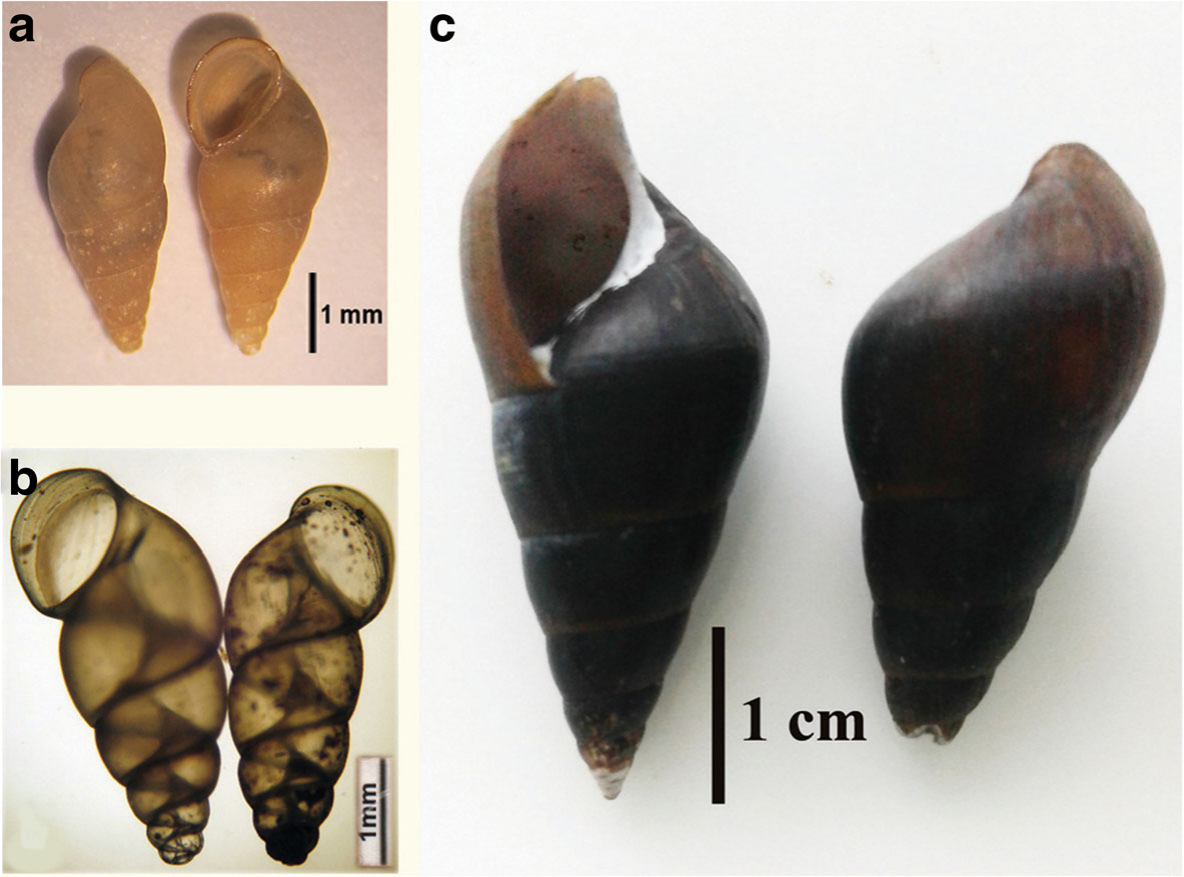
Freshwater snails infected with *Paragonimus* cercariae. **a** Triculinae sp. 1 from Yen Bai. **b** Triculinae sp. 2 from Quang Tri. **c** *Sulcospira quangtriensis* from Quang Tri

**Fig. 4:**
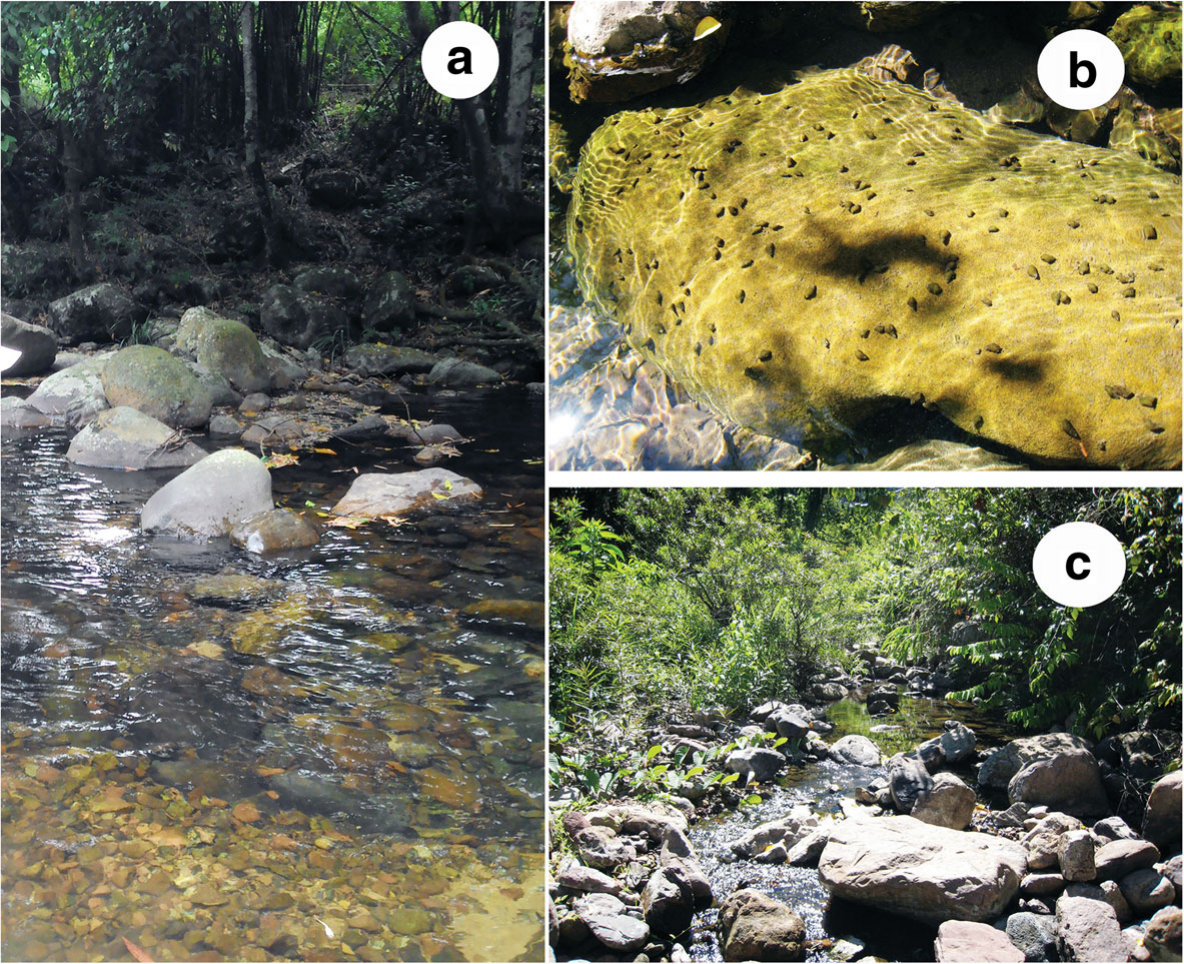
Habitats of snails in Quang Tri Province. **a** A rocky main stream with fast water flow. **b** *Sulcospira quangtriensis* on a stone of the main stream. **c** A tributary with slow water flow where *Sulcospira* and triculine snails live

The sequences obtained from five *Sulcospira* snails from Quang Tri Province were completely identical with each other. The sequences from four Triculine snails from Yen Bai Province were also identical with each other. The results of BLAST searches showed that the *cox*1 sequence of the *Sulcospira* snail from Quang Tri Province was highly similar (99%) to that of *Sulcospira quangtriensis* (FJ377265), confirming the identification of this species as *S. quangtriensis*. The *cox*1 sequences of Triculinae snails from Yen Bai and Quang Tri Provinces showed the highest similarity (but only 91% similarity) to *Gammatricula* snails (AF213342). The samples from Yen Bai and from Quang Tri Provinces differed by 12.6% and were placed separately in the phylogenetic tree (Fig. 2). The posterior probabilities of groups in which they occurred were rather low. The snail from Quang Tri was grouped with *Gammatricula songi* with a value of only 53%, the snail from Yen Bai was grouped with *Tricula wumingensis* with a posterior probability of 56%. The genus *Gammatricula* is shown as monophyletic, but with a posterior probability of only 27%. The genus *Tricula* is not monophyletic in this tree. This suggests that the snails from Yen Bai and Quang Tri Provinces belong to different genera of the subfamily Triculinae, but their taxonomic status remains uncertain. We have temporarily labelled them as Triculinae sp. 1 from Yen Bai Province, and Triculinae sp. 2 from Quang Tri Province. The snails infected with chaetomicrocercous cercariae and their habitats are shown in Figs. 3 and 4.

### Prevalence and morphology of chaetomicrocercous cercariae from snails

Infection rates of the snails with chaetomicrocercous cercariae were low, ranging between 0.07–1.0% (Table 1). In Huong Son Commune, Quang Tri Province, the density of the *S. quangtriensis* population is very high. *Sulcospira quangtriensis* collected in the rocky main stream were not positive, but those from a small tributary were infected with chaetomicrocercous cercariae.

**Table 1:** Prevalence of *Paragonimus* cercariae in snails

Location	Snail species	No. examined	No. infected (%)	*Paragonimus* species
An Lạc (Yen Bai)	Triculinae sp. 1	1200	4 (0.3)	*P. heterotremus*
	*Sulcospira* sp.	1000	0	–
	*M. tuberculata*	65	0	–
Huong Sơn (Quang Tri)	Triculinae sp. 2	1520	1 (0.07)	*P. proliferus*
	*S. quangtriensis*	500^a^	5 (1.0)	*P. westermani*
	*S. quangtriensis*	5000^b^	0	–
	*M. tuberculata*	70	0	–

^a^Collected from the small tributary

^b^Collected from the main stream

**Fig. 5:**
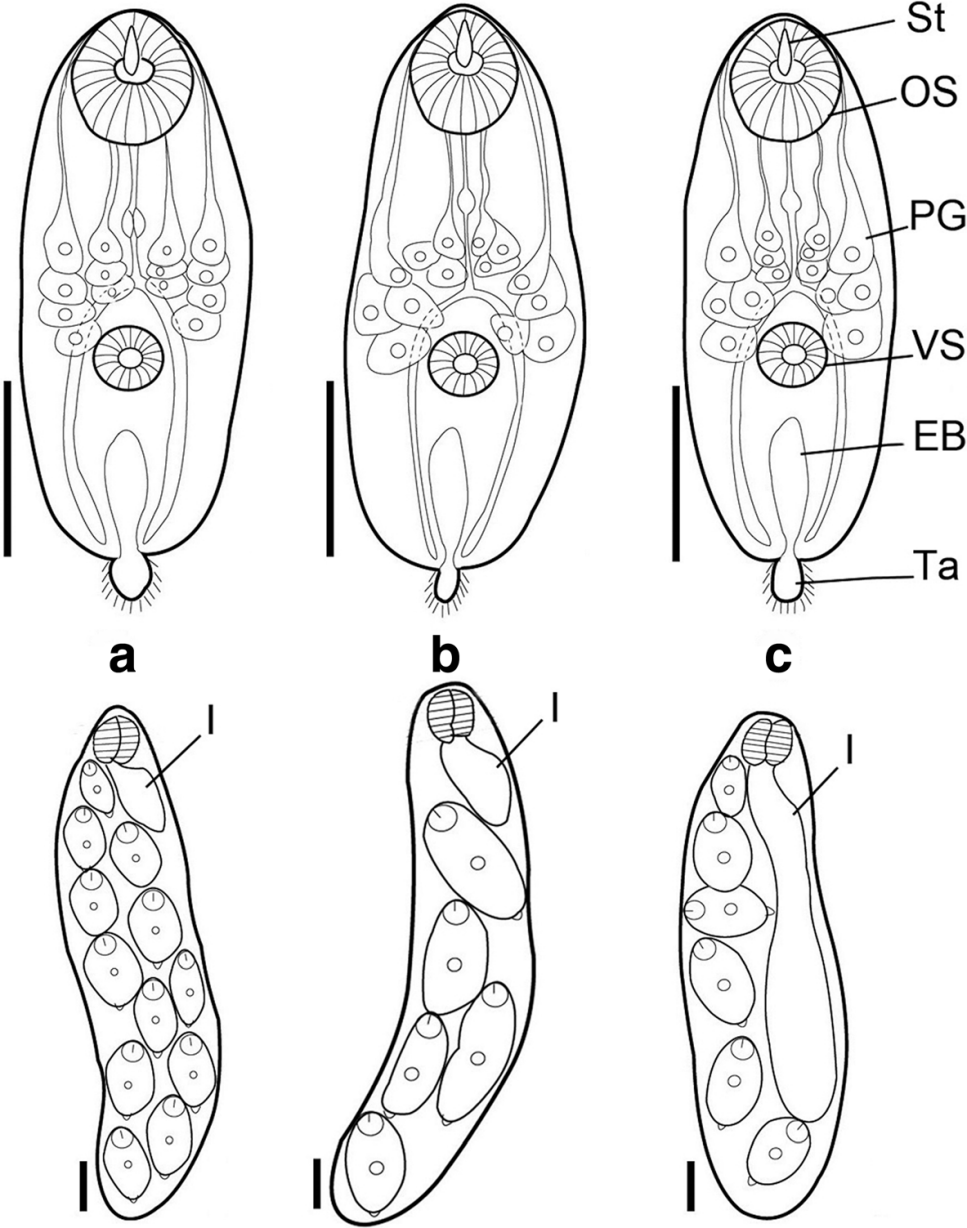
Cercariae (upper row) and rediae (lower row) of *Paragonimus* species. **a** *P. heterotremus*. **b** *P. proliferus*. **c** *P. westermani*. *Scale-bars*: 100 μm. *Abbreviations*: OS, oral sucker; VS, ventral sucker; PG, penetration gland; St, stylet; Ta, tail; EB, excretory bladder; I, intestine

The chaetomicrocercous cercariae from different snail species morphologically resemble one another (Fig. 5). In each case, the mature cercariae had a stylet and a short tail, with the measurements presented in Table 2. The body was elongated-oval in shape. The oral sucker at the anterior extremity was accompanied by a long stylet and was larger than the ventral sucker. The latter was round and situated at close to the middle of the body. A total of 14 penetration grand cells were distributed in groups of 3 and 4 cells antero-lateral to the ventral sucker. A large I-shaped excretory bladder was located between the ventral sucker and the end of the body (Fig. 5). These are typical characteristics of *Paragonimus* cercariae.

**Table 2:** Measurements (μm) of *Paragonimus* spp. larval stages collected from snails

Characteristic	*P. heterotremus*	*P. proliferus*	*P. westermani*
Cercaria			
Body	240–300 × 80–110	280–316 × 100–160	256–306 × 104–120
Oral sucker	50–70 × 50–60	60–75 × 60–75	64–80 × 64–80
Ventral sucker	28–30 × 28–30	35–50 × 35–50	40–48 × 40–48
Tail length	20–25	25–30	20–24
Stylet length	40–50	40–50	40–48
Redia			
Body	900–980 × 200–260	1000–1100 × 220–300	860–960 × 200–256
Pharynx	70–86 × 70–86	80–100 × 80–100	64–80 × 64–80
Intestine length	180–240	200–230	620–640
Intestine/body length (%)	20	20	75
No. of cercariae per redia	10–12	5–6	6–8

In contrast, the daughter rediae collected from different snail species differed from one another in the length of the intestine and the number of cercariae per redia (Fig. 5). The rediae collected from *S. quangtriensis* possessed a long intestine (about 75% of the body length) and each redia contained 6–8 cercariae. The rediae from Triculinae sp. 1 and Triculinae sp. 2 each had a short intestine (about 20% of the redia body length). Each redia from Triculinae sp. 1 contained 10–12 cercariae, double the number (5–6) seen in each redia from Triculinae sp. 2 (Fig. 5).

### Molecular identification of chaetomicrocercous cercariae

The result of BLAST searches showed that *ITS*2 sequences of cercariae from snails Triculinae sp. 1 (4 identical sequences from 4 infected snails), Triculinae sp. 2 (1 infected snail) and *S. quangtriensis* (5 identical sequences from 5 infected snails) were completely (100%) identical with those of *P. heterotremus* (AB82365), *P. proliferus* (AB663672) and *P. westermani* (LC144899), respectively.

## Discussion

Previously, cercariae considered to be of *P. heterotremus* were reported from minute snails (3–4 mm) in northern provinces of Vietnam [6]. As outlined in the introduction, the identity of these snails remains unclear. They were first identified as *Oncomelania* snails [6], then named as *Pseudotricula* [9] and re-named as *Vietricula* [11]. Our results, based on molecular analyses of *cox*1 sequences, revealed that the minute Triculinae sp. 1 snails infected with *P. heterotremus* from Yen Bai Province was related to *Tricula*, and Triculinae sp. 2 snails infected with *P. proliferus* from Quang Tri Province were related to the genus *Gammatricula.* These might be new snail species. Further detailed studies on anatomy, biology, systematics and genetics are required to confirm the identities and taxonomic status of triculine snails from Vietnam. The remaining snail species, infected with *P. westermani*, was clearly identified as *Sulcospira quangtriensis* from Quang tri Province.

Species of the genus *Paragonimus* are regarded as exhibiting host specificity for their snail hosts at the superfamily level. Members of the *P. westermani* complex have only been found in cerithioidean snails but not in the superfamily Rissooidea, within which are the first intermediate hosts of other species (*P. skrjabini*, *P. ohirai*, *P. miyazaki*, *P. fukienesis*, *P. kellikotti*, *P. proliferus*, *P. heterotremus*, *P. calienesis* and *P. mexicanus*) [4]. There is also regional host specificity; *P. westermani* from the Philippines and Malaysia use thiarid snails while those from Japan, Korea, China and Taiwan exploit pleurocercid snails as the first intermediate hosts [2–4]. In Sri Lanka, *P. westermani* cercariae were reported from snail *Paludomus* sp. of the family Paludomidae, also belonging to the superfamily Cerithioidea [19]. In the present study in Vietnam, based on molecular and morphological analyses, cercariae of three *Paragonimus* species were found, each from a different snail species. Cercariae of *P. westermani* were found from *S. quangtriensis* of the superfamily Cerithioidea, while *P. heterotremus* and *P. proliferus* were found from minute freshwater triculine snails of the superfamily Rissooidea, reinforcing the idea of host specificity at the superfamily level. The snails *Sulcospira quangtriensis* and Triculinae sp. 2 live in the same water body, but were infected with different *Paragonimus* species, *P. westermani* and *P. proliferus*, respectively. Also, cercariae of *P. heterotremus* were found from a different snail genus/species. These may suggest host specificity at the genus/species level. The unidentified *Sulcospira* sp. in Yen Bai Province may act as an intermediate host of *P. westermani* there, and Triculinae sp. 1 may be present somewhere in Quang Tri Province to maintain the life-cycle of *P. heterotremus*, of which eggs have been found in wild cats in this area [20].

The infection rates of snails with *Paragonimus* cercariae were low, between 0.07–1.0%, although those in crab hosts in the same study area were very high, between 70–100% [8]. This situation is also found in other countries, such as Malaysia, Japan and China [21–23], and helps to explain why natural first intermediate hosts are known for only a few common species, such as *P. westermani*, *P. heterotremus* and *P. ohirai* [1]. In this study, we identified the natural first intermediate hosts of three species, *P. westermani*, *P. heterotremus* and *P. proliferus*, in Vietnam*.* Among these, the first two species are common and pathogenetic to humans while *P. proliferus* is a rare species with a low infection rate in crab hosts in Huong Son Commune [8]. In China, the intermediate host of *P. proliferus* was identified as *Tricula* in experimental infection studies, but the taxonomy of these snails remains confused [1]. Our data clearly show that the natural first intermediate host of *P. proliferus* is a triculine snail, maybe *Gammatricula* or a closely related taxon.

Morphologically, cercariae of all *Paragonimus* species, where known, resemble one another closely [1, 2]. Our findings agree, with cercariae of the three *Paragonimus* species found in this study morphologically indistinguishable. However, they can be distinguished at their redial stages by the length of the intestine and the number of cercariae per redia. These may be useful characteristics for species identification of larval stage of *Paragonimus* spp. in snail hosts.

## Conclusions

The natural first intermediate hosts of three *Paragonimus* species were confirmed based on the molecular and morphological identification of both cercariae and snail hosts. Freshwater snails *Sulcospira quangtriensis*, Triculinae sp. 1 and Triculinae sp. 2 serve as snail hosts for *P. westermani*, *P. heterotremus* and *P. proliferus*, respectively, suggesting host specificity at the genus/species level. It is difficult to identify cercariae to species based on morphology, but the length of the intestine of the redia and the number of cercariae per redia are valuable characteristics for differentiation between species.

## Abbreviations

BLAST: Basic Local Alignment Search Tool
*cox*1: Mitochondrial cytochrome *c* oxidase 1
GTR: General time reversible model
ITS2: Internal transcribed spacer 2
PCR: Polymerase chain reaction
